# College of Surgeons: Adjudication of the Jacksonian Prize

**Published:** 1838-10

**Authors:** 


### COLLEGE OF SURGEONS.—ADJUDICATION OF THE JACKSONIAN PRIZE.

The Jacksonian prize for the year 1837 has been adjudged to Mr. Samuel Gaskell, of the Royal Infirmary, Manchester, for a "Dissertation on the Nature and Processes of Suppuration and Ulceration."

